# Venous Thromboembolism and Cerebrovascular Events in Patients with Giant Cell Arteritis: A Population-Based Retrospective Cohort Study

**DOI:** 10.1371/journal.pone.0149579

**Published:** 2016-02-22

**Authors:** Alberto Lo Gullo, Matthew J. Koster, Cynthia S. Crowson, Ashima Makol, Steven R. Ytterberg, Antonino Saitta, Carlo Salvarani, Eric L. Matteson, Kenneth J. Warrington

**Affiliations:** 1 Department of Clinical and Experimental Medicine, University of Messina, Messina ME, Italy; 2 Department of Medicine, Division of Rheumatology, Mayo Clinic College of Medicine, Rochester, Minnesota, United States of America; 3 Department of Health Sciences Research, Division of Biomedical Statistics, Mayo Clinic College of Medicine, Rochester, Minnesota, United States of America; 4 Department of Health Sciences Research, Division of Epidemiology, Mayo Clinic College of Medicine, Rochester, Minnesota, United States of America; 5 Department of Internal Medicine, Rheumatology Unit, Azienda Ospedaliera ASMN, Reggio Emilia, Italy; IIBB-CSIC-IDIBAPS, SPAIN

## Abstract

**Objective:**

To investigate the incidence of venous thromboembolism (VTE) and cerebrovascular events in a community-based incidence cohort of patients with giant cell arteritis (GCA) compared to the general population.

**Methods:**

A population-based inception cohort of patients with incident GCA between January 1, 1950 and December 31, 2009 in Olmsted County, Minnesota and a cohort of non-GCA subjects from the same population were assembled and followed until December 31, 2013. Confirmed VTE and cerebrovascular events were identified through direct medical record review.

**Results:**

The study population included 244 patients with GCA with a mean ± SD age at diagnosis of 76.2 ± 8.2 years (79% women) and an average length of follow-up of 10.2 ± 6.8 years. Compared to non-GCA subjects of similar age and sex, patients diagnosed with GCA had a higher incidence (%) of amaurosis fugax (cumulative incidence ± SE: 2.1 ± 0.9 versus 0, respectively; p = 0.014) but similar rates of stroke, transient ischemic attack (TIA), and VTE. Among patients with GCA, neither baseline characteristics nor laboratory parameters at diagnosis reliably predicted risk of VTE or cerebrovascular events.

**Conclusion:**

In this population-based study, the incidence of VTE, stroke and TIA was similar in patients with GCA compared to non-GCA subjects.

## Introduction

Giant cell arteritis (GCA) is an immune-mediated granulomatous large vessel vasculitis, primarily affecting the aorta and its major branches. Arterial inflammation resulting in intimal hyperplasia and luminal stenosis/occlusion is responsible for the cranial ischemic symptoms of headache, scalp tenderness, jaw claudication and vision loss. While ischemic strokes have been observed in patients with GCA, stroke remains uncommon in this condition [1–3]. Furthermore, it is uncertain, whether patients with GCA are at a higher risk of stroke compared to the general population.

Large population studies addressing the risk of incident stroke among patients with GCA compared to reference participants [4] or matched cohorts [5, 6] have recently reported an increased risk of stroke among patients with GCA. However, each of these studies has been limited by the use of administrative data sets in which the diagnosis of GCA and stroke were based on the presence of diagnostic coding and surrogate prednisone prescription data without review of the medical record to objectively confirm the presence of either condition. This is evidenced by either the absence of temporal artery biopsy data [5, 6] or only a minority of patients having concurrent coding for temporal artery biopsy procedures among those with diagnosis codes for GCA [4]. The likelihood of both disease exposure and outcome misclassification is further supported by the loss of significance in stroke risk when supplemental and adjusted analyses are performed [4, 5] or stricter surrogate definitions of GCA are employed [5, 6].

In addition to stroke, venous thromboembolism (VTE) has been reported to be increased among patients with systemic vasculitis. While the association between vasculitis and VTE has been most closely associated with Behçet’s disease and the antineutrophil cytoplasmic antibody (ANCA)-associated vasculitides, the relationship of GCA and VTE remains less definite. Recent studies have either been limited to inpatient only data [7] or to large administrative data sets in which both case and outcome misclassification can occur and limited disease specific information is known [7, 8].

In order to address these limitations, the objective of this study was to evaluate the risk of incident stroke and VTE in GCA among a population-based cohort with age-, sex-, and calendar-year matched referent subjects in which full access to near complete medical records was available for all study subjects allowing for objective confirmation of disease and outcome diagnoses through the use of standardized definitions.

## Patients and Methods

This was a retrospective population-based study performed using resources of the Rochester Epidemiology Project (REP) medical record linkage system [9]. The REP allows virtually complete access to medical records from all community medical providers including Mayo Clinic, Olmsted Medical Center and their affiliated hospitals, local nursing homes, and the few private practitioners in Olmsted County, MN. The uniqueness of the REP and its advantages in performing population-based studies in rheumatic diseases has been previously described [9]. The study was approved by the Institutional Review Board at Mayo Clinic, Rochester, Minnesota. At time of enrollment in the REP study, all participants provided written informed consent to allow medical records to be used for research purposes. Patients with withdrawn consent prior to the end of the study completion were not included in the final analyses.

We retrospectively reviewed the incidence cohort of patients with GCA diagnosed between January 1, 1950 and December 31, 2009 based on American College of Rheumatology 1990 GCA classification criteria [10]. We also included patients diagnosed with GCA ≥ 50 years of age with elevation of erythrocyte sedimentation rate or C-reactive protein and computed tomography angiography (CTA), magnetic resonance angiography (MRA) or positron emission tomography (PET) evidence of large vessel vasculitis involving the ascending aorta and its branches. For each patient with GCA, the population of Olmsted County of the same age and sex as the patient on the patient’s date of diagnosis was assembled. From this pool of potential comparators of the same age and sex on the same day, living in the same population, one person was randomly chosen by computer algorithm to be the comparator subject for the assigned GCA patient in the study. All subjects were followed longitudinally through all available community medical records until death, migration from Olmsted County, or December 31, 2013.

A master list of potential codes for diagnoses of VTE and cerebrovascular events was electronically cross-matched with the medical records of the patients in the GCA cohort and the non-GCA comparison cohort. The subset of patients in each cohort whose medical records contained one or more of the codes of interest were reviewed by a physician abstractor (ALG) to confirm the diagnosis based on the specified criteria for each disease entity (Table 1). VTE included DVT (definite and probable) and PE. A short period of anticoagulation therapy while awaiting completion of diagnostic evaluation for either suspected DVT or PE was not considered sufficient for inclusion as VTE. Cerebrovascular events included ischemic stroke, hemorrhagic stroke, transient ischemic attack (TIA), and amaurosis fugax. Among the non-GCA cohort, the date of first occurrence of unique VTE and cerebrovascular event was recorded. Disease prevalence was assessed by recording those cases in which a VTE or cerebrovascular event occurred prior to the diagnosis of GCA or prior to the index date in the non-GCA comparison cohort. Prevalent cases were excluded from the incidence analyses.

**Table 1:** Criteria for event inclusion in the study for venous thromboembolism and cerebrovascular events in patients with GCA and non-GCA subjects*.

Deep vein thrombosis(definite)	Verified by venography, ultrasound, or thrombus removedduring surgery or autopsy.
Deep vein thrombosis(probable)	Confirmatory tests indeterminate or not done but all the following were present: a) physician diagnosis present in medical record, b) signs and symptoms consistent with deep vein thrombosis documented in exam, and c) patient underwent therapy with anticoagulant (heparin, warfarin, or similar agent).
Pulmonary embolism	Verified by pulmonary angiography, CT/MRI angiography, ventilation-perfusion lung scan (interpreted as high probability), or thrombus removed during surgery or autopsy.
Ischemic stroke	Focal neurological deficit persisting > 24 hours, compatible with altered circulation to a limited region of brain parenchyma, diagnosed by a neurologist (CT/MRI confirmation optional).
Hemorrhagic stroke	Acute onset of focal neurologic deficit associated with headache, vomiting, altered level of consciousness, signs of meningeal irritation, and blood stained cerebrospinal fluid. Diagnosis made by neurologist. If done, CT/MRI showed parenchymal hemorrhage.
Transient ischemic attack	Transient period of ≤ 24 hours of neurologic dysfunction caused by focal brain ischemia without evidence of acute infarction on neuroimaging or clinical evidence of permanent injury or neurologic sequelae.
Amaurosis fugax	Transient monocular visual disturbance with abrupt onset and rapid resolution, lasting < 24 hours and due to altered circulation to the optic nerve.

*GCA = giant cell arteritis

CT = computed tomography; MRI = magnetic resonance imaging

Cardiovascular risk factors have previously been collected on patients in the GCA cohort and in the non-GCA cohort [11]. These included the following: age; sex; obesity; cigarette smoking status (current, former, or never); presence of dyslipidemia, hypertension, diabetes mellitus, and/or chronic kidney disease; myocardial infarction; and coronary revascularization procedures (e.g., coronary artery bypass graft, percutaneous angioplasty, insertion of stents, and atherectomy).

## Methods

Descriptive statistics (means, percentages) were used to summarize the characteristics of the two cohorts. Comparisons of baseline characteristics in the two cohorts were performed using chi-square and rank-sum tests. The cumulative incidence of VTE and cerebrovascular events in patients with and without GCA, adjusted for the competing risk of death, was estimated and compared using methods by Gray [12, 13]. Patients who died prior to experiencing VTE or cerebrovascular events were appropriately accounted for to avoid overestimation of the rate of occurrence of the event, which may have occurred if such subjects were simply censored at death. Patients with history of prior VTE or cerebrovascular events were excluded from these analyses.

Person-year methods were used to calculate rates of development of VTE and cerebrovascular events during follow-up. Rate ratios comparing GCA to non-GCA were obtained and 95% confidence intervals were calculated using Poisson regression methods. Cox proportional hazard models adjusted for age, sex and calendar year of GCA diagnosis were used to identify predictors of incident VTE and cerebrovascular events among patients with GCA. Analyses were performed using SAS version 9.3 (SAS Institute, Cary, NC, USA). P-values < 0.05 were considered significant.

## Results

### Demographics

The original cohorts included 245 patients with GCA and 245 matched non-GCA subjects. Prior to study completion 6 patients (1 GCA; 5 non-GCA) declined use of their medical records for research purposes per Minnesota law and were not included in the final analyses. The GCA cohort therefore consisted of 244 subjects who were diagnosed with GCA (89% biopsy-proven) between January 1, 1950 and December 31, 2009. There were 193 (79%) women and 51 men with a mean ±SD age at diagnosis of 76.2 ± 8.2 years. All subjects were residents of Olmsted County, Minnesota at the time of GCA diagnosis and during study follow-up. The mean length of follow-up was 10.2 ± 6.8 years. The 244 patients with GCA were compared to 240 subjects without GCA of similar age and sex who were also residents of Olmsted County, Minnesota at the index date. The non-GCA cohort consisted of 190 (79%) women and 50 men, with a mean age of 75.8 ± 8.5 years and a mean follow-up of 10.8 ± 7.9 years. The GCA cohort was followed for a total of 2,480 person-years, while the non-GCA cohort was observed for 2,588 person-years. Baseline characteristics of the two cohorts are shown in Table 2. At index date, non-GCA subjects were more frequently treated with a lipid lowering medication (16.5% non-GCA versus 9.6% GCA; p = 0.027) and a higher percentage had diabetes mellitus (16.7% non-GCA versus 6.6% GCA; p = 0.001). Patients with GCA were more likely to be on aspirin at time of diagnosis than non-GCA subjects (22.4% GCA versus 14.5% non-GCA; p = 0.028). Comprehensive anti-coagulant data was only available for patients from 1997–2009 (n = 83 GCA, n = 86 non-GCA). Among this patient subset, no significant difference in anti-coagulant use at time of GCA diagnosis/index date was observed (4.8% GCA versus 11.6% non-GCA; p = 0.11).

**Table 2:** Baseline characteristics of 244 patients with incident GCA in the period 1950 to 2009 compared with 240 subjects without GCA.

Characteristic (%)	GCA(N = 244)	Non-GCA (N = 240)	P value
Age, years*	76.2 (8.2)	75.8 (8.5)	0.74
Female	193 (79.1)	190 (79.2)	0.99
Follow up duration, years*	10.2 (6.8)	10.8 (7.9)	-----
Body mass index*	25.2 (5.1)	26.0 (5.3)	0.094
Smoking, ever	96 (42.3)	101 (44.7)	0.61
Systolic blood pressure, mm Hg*	139.8 (20.5)	140.8 (20.3)	0.76
Diastolic blood pressure, mm Hg*	76.0 (11.1)	76.2 (12.1)	0.96
Antihypertensive medication	106 (44.2)	125 (52.5)	0.068
Aspirinǂ	52 (22.4)	34 (14.5)	0.028
Total cholesterol*	209.1 (49.3)	207.5 (42.3)	0.88
Low density lipoprotein*	113.1 (36.1)	108.0 (31.8)	0.45
Lipid lowering medication	23 (9.6)	39 (16.5)	0.027
Atrial fibrillation	31 (13.0)	27 (11.0)	0.62
Chronic kidney disease	12 (5.0)	7 (3.0)	0.26
Diabetes mellitus	16 (6.6)	40 (16.7)	0.001

Of the original cohort of 245 patients with GCA and 245 non-GCA subjects, 6 patients (1 GCA; 5 non-GCA) withdrew research authorization before study completion and were removed from final analyses.

*Mean (±SD).

^ǂ^ = 232 GCA and N = 234 Non-GCA

Prevalent cases of VTE and cerebrovascular events for patients with GCA and matched non-GCA subjects are listed in Table 3. No significant differences in prevalent events were noted among the VTE or cerebrovascular event subsets. The median (interquartile range [IQR]) time from VTE to GCA diagnosis was 5.1 (1.0, 21.7) years compared to 10.0 (1.5, 27.7) years from VTE to index date among non-GCA subjects (p = 0.24). Median (IQR) time of stroke event prior to GCA diagnosis/index date was 3.4 (3 days, 27.0) years in the GCA cohort and 2.1 (5 days, 17.3) years in the non-GCA subjects (p = 0.62). In the six months prior to GCA diagnosis/index date, four strokes occurred in the GCA cohort and three strokes in the non-GCA subjects. No VTE events occurred during the preceding six months in either group.

**Table 3:** Cumulative incidence rates of venous thromboembolism and cerebrovascular events in the 244 patients with incident GCA in the period from 1950–2009 compared to the 240 subjects without GCA.

	No. of events prior to incidence or index date		No. of events after incidence or index date		Cumulative incidence ± SE (%) at 10 years*		
Event	GCA	Non-GCA	GCA	Non-GCA	GCA	Non-GCA	p-value**
Deep vein thrombosis	7	6	12	5	4.8 ± 1.4	1.8 ± 0.9	0.08
Pulmonary embolism	4	4	11	7	3.5 ± 1.2	2.7 ± 1.1	0.34
Venous thromboembolism	10	8	16	10	6.3 ± 1.6	3.7 ± 1.3	0.22
Stroke (combined)	11	13	28	28	8.5 ± 1.9	9.2 ± 2.0	0.94
Ischemic stroke	10	12	25	23	7.1 ± 1.7	6.4 ± 1.7	0.67
Hemorrhagic stroke	1	1	4	8	1.3 ± 0.8	2.6 ± 1.1	0.25
Transient ischemic attack	7	4	6	6	2.3 ± 1.0	1.7 ± 0.8	0.97
Amaurosis fugax	3	4	6	0	2.1 ± 0.9	0	0.014
Any cerebrovascular eventǂ	18	18	30	27	9.6 ± 2.0	8.5 ± 1.9	0.58

^ǂ^ Stroke (ischemic or hemorrhagic), transient ischemic attack or amaurosis fugax

*Cumulative incidence is adjusted for the competing risk of death.

**Calculated using methods by Gray

### Venous Thromboembolism

**Fig 1:**
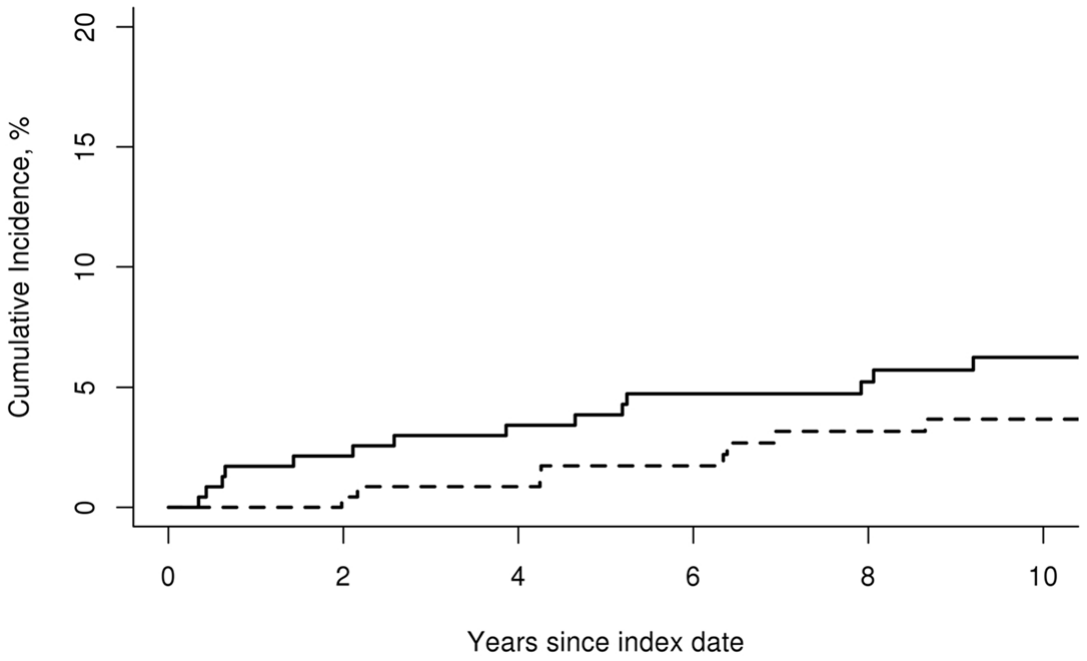
Cumulative incidence of venous thromboembolic events. Cumulative incidence (%) of venous thromboembolism in 244 patients with incident GCA in the period 1950–2009 (solid line) compared to 240 subjects without GCA (dashed line).

During follow-up in the GCA cohort, 16 incident VTE events (6 DVT alone, 4 PE alone, 6 DVT and PE) occurred. Ten incident VTE events (3 DVT alone, 5 PE alone, 2 DVT and PE) were diagnosed within the non-GCA comparison cohort during the same time period. The cumulative incidence (%) of VTE at 10 years was similar between patients with GCA and non-GCA subjects (cumulative incidence ± standard error (SE); 6.3 ± 1.6 versus 3.7 ± 1.3, respectively; p = 0.22) [Fig 1]. The overall rate of venous thromboembolic events was 6.6 per 1,000 person-years in the GCA cohort and 3.9 per 1,000 person-years in the non-GCA cohort [rate ratio (95% confidence interval [CI]); 1.64 (0.77, 3.74)].

### Cerebrovascular events

Among patients diagnosed with GCA, 30 incident cerebrovascular events occurred (21 stroke alone, 4 amaurosis fugax alone, 3 TIA and stroke, 1 amaurosis fugax and TIA, 1 amaurosis fugax, 0 TIA and stroke) compared to 27 incident cerebrovascular events within the non-GCA subjects (1 TIA alone, 23 stroke alone, 0 amaurosis fugax, 3 TIA and stroke). For ischemic strokes, the territory of vascular involvement was recorded and compared between patients with GCA and non-GCA subjects. No difference in vascular territory (i.e., anterior versus posterior circulation) was observed.

**Fig 2:**
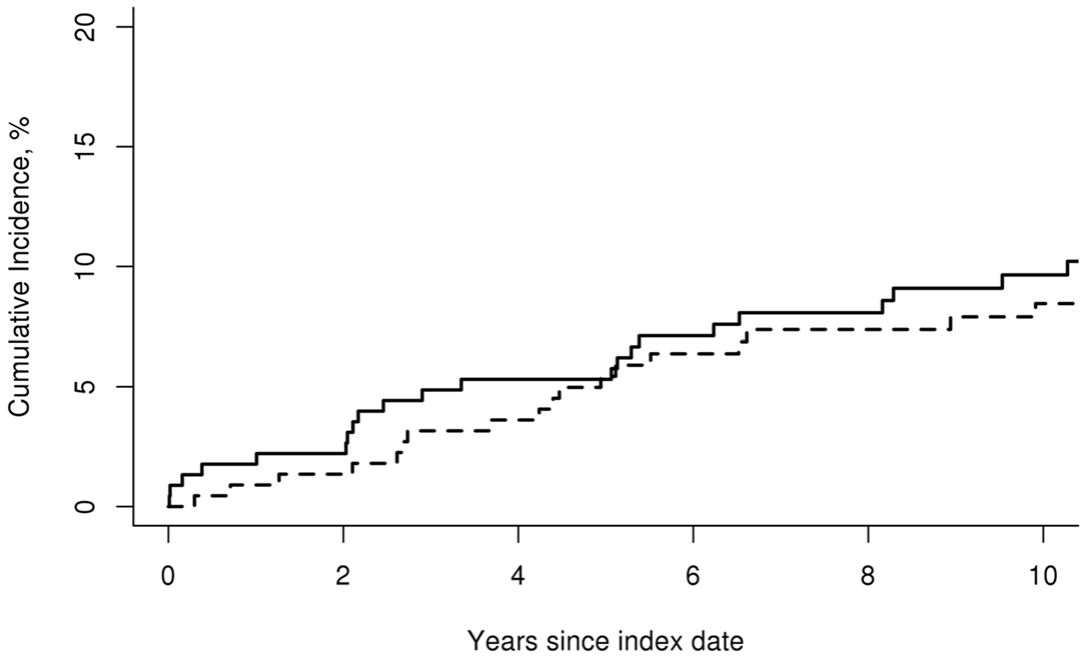
Cumulative incidence of cerebrovascular events. Cumulative incidence (%) of cerebrovascular events in 244 patients with incident GCA in the period 1950–2009 (solid line) compared to 240 subjects without GCA (dashed line).

The cumulative incidence (%) of cerebrovascular events at 10 years was also similar between patients with GCA and non-GCA subjects (cumulative incidence ± SE; 9.6 ± 2.0 versus 8.5 ± 1.9, respectively; p = 0.58) [Fig 2]. Evaluation of the cumulative incidence (%) at 10 years for the cerebrovascular event subtypes did not show a difference for ischemic stroke, hemorrhagic stroke or TIA (Table 3). Patients with GCA, however, had a higher 10-year cumulative incidence (%) of amaurosis fugax compared to non-GCA subjects (cumulative incidence ± SE; 2.1 ± 0.9 versus 0, respectively; p = 0.014). The overall rate of cerebrovascular events was 13.0 per 1,000 person-years for patients with GCA and 11.0 per 1,000 person-years for non-GCA subjects [rate ratio (95% CI); 1.18 (0.70, 1.99)].

### Incidence rates

Mean age at first event did not differ between patients with GCA and non-GCA subjects for VTE (81.3 versus 85.9 years; p = 0.16) or cerebrovascular event (80.6 versus 83.8 years; p = 0.30).

### Risk Factors

There were no significant differences in baseline characteristics, clinical presentation features, laboratory parameters, or diagnostic examinations comparing patients with GCA to non-GCA subjects presenting with VTE or cerebrovascular event. Additionally, patients with GCA who developed VTE or a cerebrovascular event had no increase in mortality compared to non-GCA subjects experiencing the same event.

Evaluation of incident VTE and/or cerebrovascular events among patients with GCA did not show any baseline GCA symptoms, laboratory parameters, comorbidities, or medication use to be significantly associated with an increased risk for VTE or cerebrovascular events during the follow-up period with adjustment for age, sex, and calendar year of GCA diagnosis (Table 4). No association was found between VTE or cerebrovascular events and GCA status after adjusting for age, sex, calendar year of GCA diagnosis, body mass index, medication use (antihypertensive, lipid lowering, aspirin) and presence of diabetes mellitus. The adjusted hazard ratios for incident VTE and cerebrovascular events among patients with GCA compared to non-GCA subjects were 1.48 (95% CI: 0.65, 3.35; p = 0.35) and 1.37 (95% CI: 0.80, 2.27; p = 0.26), respectively. Additional adjusted analysis comparing events among only the biopsy-proven GCA cases to non-GCA subjects similarly revealed nonsignificant differences for VTE (1.60 [95% CI: 0.72, 3.55; p = 0.25]) and cerebrovascular events (1.17 [95% CI: 0.66, 2.07; p = 0.59]).

**Table 4:** Baseline risk factors in patients with incident giant cell arteritis during the period 1950–2009 and association with time to first cerebrovascular or venous thromboembolism event.

Variable	Cerebrovascular Event HR* (95% CI)	P-value	Venous Thromboembolism HR* (95% CI)	P-value
TAB positive	0.86 (0.25, 2.97)	0.81	2.02 (0.26, 15.93)	0.51
Fever > 100°F	1.32 (0.52, 3.38)	0.56	0.31 (0.04, 2.53)	0.27
Weight loss > 5 kg	1.19 (0.48, 2.96)	0.71	1.02 (0.22, 4.74)	0.98
Headache	0.84 (0.36, 1.95)	0.68	0.69 (0.19, 2.41)	0.56
Jaw claudication	0.67 (0.30, 1.50)	0.33	1.32 (0.39, 4.44)	0.65
Scalp tenderness	1.56 (0.70, 3.48)	0.28	0.80 (0.22, 2.88)	0.73
Tender temporal artery	2.23 (0.98, 5.06)	0.055	1.88 (0.48, 7.33)	0.36
Transient visual loss	1.06 (0.14, 7.91)	0.96	0.00 (0.00, 0.00)	0.99
Permanent visual loss	3.80 (0.47, 30.47)	0.21	5.55 (0.64, 48.46)	0.12
PMR symptoms	1.65 (0.76, 3.58)	0.20	1.65 (0.48, 5.68)	0.43
Hemoglobin, g/dL	1.30 (0.91, 1.85)	0.15	1.23 (0.69, 2.21)	0.48
WBC, x 10^9^/L	0.99 (0.98, 1.01)	0.27	1.00 (0.97, 1.02)	0.76
ESR, mm/h	1.00 (0.98, 1.01)	0.70	0.99 (0.97, 1.01)	0.20
Smoking	1.15 (0.50, 2.64)	0.74	1.39 (0.46, 4.22)	0.56
Hypertension	1.23 (0.51, 2.99)	0.64	3.17 (0.84, 12.05)	0.09
Diabetes mellitus	2.08 (0.27, 15.97)	0.48	0.00 (0.00, 0.00)	0.99
Coronary artery disease	1.70 (0.59, 4.93)	0.33	3.00 (0.74, 12.16)	0.13
Hyperlipidemia	1.60 (0.65, 3.94)	0.31	1.61 (0.41, 6.37)	0.50

HR = Hazard ratio; CI = confidence interval; TAB = temporal artery biopsy; WBC = white blood cell count; ESR = erythrocyte sedimentation rate

*adjusted for age, sex and calendar year of GCA diagnosis

## Discussion

Chronic inflammatory diseases including rheumatoid arthritis [14, 15], systemic lupus erythematosus [16–18], inflammatory bowel disease [19, 20], granulomatosis with polyangiitis [21–23], and Behçet’s disease [24, 25] have been strongly associated with an increased risk of vascular thrombotic or thromboembolic disease. The risk of incident venous thromboembolism and cerebrovascular events in patients with GCA, however, is less well defined. To our knowledge this is the first population-based study to provide the incidence of VTE and cerebrovascular events among patients with GCA and matched comparator subjects where standardized case definitions and comprehensive medical record data were available to verify both GCA and event diagnoses.

### Visual ischemic events

Visual ischemic complications are frequently described presenting symptoms of GCA. Diplopia, transient visual loss (amaurosis fugax), and permanent visual loss have been described in 14–26% of patients with GCA [1, 26–30]. As anticipated, our study showed an increased incidence of amaurosis fugax in patients with GCA, likely due to the frequent arteritic involvement of the ophthalmic and posterior ciliary arteries associated with this disease process [31]. This is in contrast to the general population, where amaurosis fugax is most commonly a symptom of atherosclerotic carotid artery disease [32].

### Stroke and transient ischemic attack

The exact frequency of cerebrovascular events in GCA has been difficult to define as the timing of stroke and TIA onset with GCA diagnosis has varied among publications. Among descriptive cohorts evaluating CVA, 1.5–7.5% of patients with GCA were found to develop stroke or TIA by the time of diagnosis or within 4 weeks following treatment initiation [1, 26, 28–30, 33–35]. Population-based stroke registries have also been utilized to estimate the frequency of stroke in patients with GCA. Samson et al. reviewed 2,305 patients with strokes and found 57 (2.4%) patients with a history of biopsy-proven GCA; four (7%) of which were determined to have had a GCA-related stroke. The overall incidence rate of GCA-related stroke was 0.76/100,000/year [2]. Wiszniewska and colleagues, however found a much lower frequency using the Swiss Lausanne Stroke Registry where only six (0.15%) of the 4,086 confirmed first-ever strokes were associated with biopsy confirmed GCA [3].

An increased inflammatory burden and propensity toward a hypercoagulable state has been hypothesized as the reason for an increased incidence of vascular events in patients with autoimmune diseases. The direct influence of inflammation and hypercoagulability on developing cranial vascular events in GCA has been debated. Indeed, several studies, including ours, have failed to predict an increased risk of stroke or TIA in patients with GCA when evaluating baseline inflammatory markers or the presence of constitutional symptoms [29, 34–36]. Furthermore, others have noted a lower inflammatory response at GCA diagnosis to confer a higher risk of ischemic complications; hypothesizing that inflammation induced angiogenesis may counteract the development of stroke in GCA [1, 26–28, 37–39]. On the other hand, traditional cardiovascular risk factors, including age [29], smoking [40], hypertension [3, 28, 34, 40], and hyperlipidemia [34], have been frequently noted to confer a higher risk of CVA in patients with GCA.

Analysis of the risk of stroke and TIA in GCA after adjustment for cardiovascular risk factors has been hindered by a lack of population-based case-control studies. In fact, only three such studies have been performed prior to our report, all of which relied on administrative databases and diagnostic coding. Ray and colleagues used the Ontario, Canada health care database to compare cardiovascular disease between newly diagnosed GCA patients, patients with osteoarthritis, and unaffected controls [4]. While composite cardiovascular disease was significantly elevated on both crude and adjusted analyses, stroke was not significantly increased in patients with GCA compared to osteoarthritis or unaffected controls when adjusted for age, sex and baseline co-morbidities.

Tomasson et al. similarly performed an observational cohort study using the United Kingdom primary care database to assess cardiovascular disease in patients with GCA [5]. These authors found the incidence rate of CVA to be higher in patients with incident GCA than referent subjects (8.0 events versus 6.3 events per 1,000 person-years, respectively). However, patients with GCA were more frequently smokers and hypertensive as well as taking beta-blockers, statins, nitrates, and anti-platelet medications at baseline. When adjusted for baseline medications, CVA risk was no longer significantly elevated.

Finally, in a Canadian matched cohort study, Amiri et al. observed patients with GCA (n = 809) to have an increased risk of stroke compared to age-, sex-, and entry-time matched non-GCA (n = 8577) cases [6]. In this study, GCA cases were identified by associated ICD-9/ICD-10 coding in addition to at least one prescription for oral glucocorticoids between one month before and six months after the index date. Stroke on the other hand, was identified purely by ICD-9/ICD-10 coding. While a fully adjusted primary analysis demonstrated a significantly increased risk of stroke among patients with GCA [HR 2.04 (1.43, 2.93)], a sensitivity analysis using a stricter definition of GCA (i.e. 5 or more glucocorticoid prescriptions) failed to show a significantly higher risk of stroke between the two cohorts [HR 1.37 (0.96, 1.96)].

In our cohort, we did not observe an increased risk of stroke or TIA in patients with GCA. While our results differ from those of other studies, reasons for such may include use of standard definitions and direct access to medical records for comprehensive review and validation of both the disease diagnosis, and the outcomes of interest. While risk factors at baseline differed between the two cohorts with non-GCA subjects having a higher frequency of diabetes mellitus and treatment with lipid lowering medications as well as a lower frequency of aspirin use at time of diagnosis, when adjusted for these and additional variables the risk of stroke remained similar between the groups.

### Venous thromboembolism

Although several studies have addressed the risk of cerebrovascular events in GCA, few have focused on the risk of VTE. Our cohort is the first study to evaluate patients with physician confirmed GCA to age-, sex-, and calendar-year matched population-based comparator subjects using standardized case definitions. In our study we did not observe an increased risk of DVT, PE, or composite VTE between patients with GCA and non-GCA subjects.

An observational cohort study using the British Columbia administrative health database by Aviña-Zubieta et al. identified a near 4-fold increased risk of both DVT and PE in patients with GCA compared to age and sex matched controls [8]. The GCA patients in that study, however, had a higher frequency of hypertension, greater Charlson co-morbidity index, and more frequent hospitalizations over the study period; characteristics which either alone or in combination could increase the risk of thromboembolism. Recently, Unizony and colleagues utilized the United States Nationwide Inpatient Sample database to study a total of 8.2 million patients admitted for pneumonia, myocardial infarction, ischemic stroke, and femoral neck fracture [7]. Among the 9311 patients with a documented diagnosis of GCA on admission, both DVT (1.5% versus 0.7%) and PE (0.9% versus 0.6%) were increased compared to non-GCA patients. The GCA patients were notably older (80 versus 74 years) and more frequently female (76% versus 53%).

Database studies, while useful to evaluate large populations, are limited by a reliance on diagnostic coding without medical record availability to confirm disease or event occurrence. Indeed neither of the above studies had temporal artery biopsy or ACR criteria available for confirmation of GCA diagnosis and they were unable to confirm VTE diagnosis by documented physical examination or radiology, as we were able to perform in our study. Results from Bhavsar and colleagues further confirm this discrepancy by noting VTE was not increased in their multi-center study following 256 GCA patients over a mean observation of 3.2 years. In fact, no incident events occurred during their follow-up period. [41].

Strengths of the present study include the use of a population-based incidence cohort with long-term follow-up and a complete medical record, permitting the collection of information available for all subjects. With the exception of a higher proportion of the working population employed in the healthcare industry, and correspondingly higher education levels, on the whole, results of this study using the population of Olmsted County are generalizable to the populations of interest elsewhere [9]. Limitations of our study are those inherent to the retrospective design. Only those persons who had a medical encounter during a VTE or CVA event were identified and included. While some VTE or cerebrovascular events that happened outside Olmsted County, MN may not have been included, we expect this occurrence to be rare and equal among both study groups. Additionally, given VTE and cerebrovascular events occur with relatively low overall frequency in patients with GCA, it is possible that our limited population size was not sufficient to identify a small increased risk effect. It is also possible our comparator group may have been at increased risk for events compared to the GCA cohort, due to the higher proportion of patients with risk factors among the comparators, which could decrease the relative risks. However, when adjusted analyses were performed including variables that differed between the groups at baseline, no change in relative risk was observed. Anti-coagulant use was not significantly different between groups; however, complete data was only available for a subset of patients. If percentage of anti-coagulant use among this subset were extrapolated to the entire cohort, patients with GCA would actually have a significantly lower proportion of anti-coagulant use compared to the non-GCA group (p = 0.008). Therefore, in this study, it is unlikely that anti-coagulant use contributes to the lack of a detectable increased risk of VTE or cerebrovascular events among GCA patients. Finally, patients in the early decades of this cohort diagnosed prior to the availability of routine cranial imaging for stroke may have a potential increased risk of misclassification if cerebrovascular event was made on clinical grounds alone. However, since both the cases and comparator subjects are from the same population, it is anticipated if such misclassification did occur this would be equal among both groups evaluated and would not affect the overall relative risk of these events.

In conclusion, in this population-based cohort, patients with GCA were at an increased risk for amaurosis fugax; however, the risk of VTE, stroke and TIA was similar to non-GCA subjects. Further population-based studies with control subject comparison are needed to confirm these findings.
